# Indigenous microbiology: Why Indigenous knowledge matters in microbial science

**DOI:** 10.1093/femsmc/xtag024

**Published:** 2026-05-06

**Authors:** Francyeli Araújo Silva, Ruthchelly Tavares da Silva, Nikki Harcourt, Eva Biggs, Marciane Magnani

**Affiliations:** Laboratory of Microbial Process in Foods, Department of Food Engineering, Federal University of Paraíba, João Pessoa 58051-900, Brazil; Laboratory of Microbial Process in Foods, Department of Food Engineering, Federal University of Paraíba, João Pessoa 58051-900, Brazil; New Zealand Bioeconomy Science Institute, Hamilton 7608, New Zealand; New Zealand Bioeconomy Science Institute, Lincoln 7608, PO Box 69040, New Zealand; Laboratory of Microbial Process in Foods, Department of Food Engineering, Federal University of Paraíba, João Pessoa 58051-900, Brazil

**Keywords:** microbiome research, traditional foods, environmental sustainability, human health

## Abstract

Indigenous knowledge has long engaged with microorganisms through relationships with environment, food, and health, yet these understandings remain marginal in mainstream microbiology. In this article, we argue that Indigenous microbiology offers a broader framework for microbial science by placing microorganisms within ecological, cultural, and ecosystem-specific relationships. This perspective is relevant to current challenges in sustainable agriculture, food security, and antimicrobial resistance. Drawing on examples from environmental management, traditional fermented foods, and therapeutic practices, we show that Indigenous knowledge can deepen microbiome research and contribute to developing healthy and sustainable strategies. Indigenous microbiology should not be treated as an informal add-on to existing science, but as a valuable framework for asking better questions about microbial life and its role in human and environmental wellbeing.

## Introduction

Recent research shows that Indigenous perspectives broaden the scope of microbiology by viewing microbes as part of relational networks that connect humans, other living systems, land, and water (Robinson et al. 2022). The emerging field of Indigenous microbiology brings together ancestral knowledge and contemporary microbial science, offering insights into ecosystem sustainability, traditional health practices, and the role of microorganisms in cultural heritage. Although the field is not yet formally defined, growing literature demonstrates its relevance to areas such as food fermentation, environmental stewardship, and biotherapeutics (Warbrick et al. 2023).

Indigenous knowledge is grounded in generations of observation and practical engagement with the environment. Concepts such as ‘indigenous microorganisms’ in environmental biotechnology similarly recognize the importance of locally adapted microbial communities in processes, including bioremediation, waste treatment, and sustainable agriculture (Kumar and Sai Gopal 2015). These perspectives align with land-based Indigenous practices and support microbiome research that is more ecosystem-specific and ecologically grounded.

Interest in these perspectives is increasing as microbiome science expands. Indigenous communities have long recognized connections between environmental systems, human health, and microbial relationships that Western science has only recently formalized through frameworks such as One Health (Bader et al. 2023). Incorporating Indigenous perspectives may therefore strengthen microbiome research while supporting more culturally relevant health initiatives and addressing persistent health inequities (Ogunrinola et al. 2020, Silk et al. 2024).

Research comparing microbial diversity across populations highlights the importance of environmental and lifestyle factors. Indigenous or traditionally living communities often exhibit greater microbial diversity than populations in industrialized settings, with shifts towards westernized lifestyles associated with changes in microbiome composition (Handsley-Davis et al. 2023). For example, Me’phaa Indigenous children in rural Mexico show higher gut microbial diversity and greater abundance of VANISH (volatile or associated negatively with industrialized societies of humans) taxa compared with urban Mexican children, whose microbiomes are dominated by taxa linked to urbanization and modern diets (Sánchez-Quinto et al. 2020). Socioeconomic context can also influence microbiome composition, as shown in studies of Arab Indigenous communities where infant gut microbiomes differed across villages with varying socioeconomic conditions (Lapidot et al. 2022).

These patterns also provide insight into current societal shifts towards organic, minimally processed, and sustainability-oriented food systems. While often framed as modern innovations, such approaches may partially reflect ancestral exposure patterns characterized by greater microbial diversity across the food chain. Diets based on less processed foods and low-input production systems tend to preserve microbial complexity, which has been associated with beneficial host–microbiome interactions (Bertola et al. 2021, Kotsiliti 2026).

Complementing this perspective, a co-evolutionary framework provides an additional way to understand interactions between humans and microbiota, particularly in the context of traditional food systems and environmental practices (Ma et al. 2023). Humans have evolved in continuous interaction with microbial communities associated with food, soil, water, and natural environments. This long-term exposure has shaped host physiology, including immune function and metabolism, supporting the view that human–microbiome relationships are dynamic and co-adaptive (Ojeda-Linares et al. 2024).

Together, these findings highlight the importance of understanding microbial systems within broader environmental and cultural contexts. Indigenous microbiology provides a framework for doing so by linking microbial science with ecosystem-specific knowledge and long-standing ecological relationships. In this opinion piece, we explore how Indigenous knowledge can inform microbiome research across three interconnected domains: environmental systems, traditional food practices, and human health.

## Environmental relationship with native practices

Indigenous communities have long recognized the central role of microorganisms in environmental processes. Microbial life represents most of Earth’s biodiversity and underpins key ecological functions, including nutrient cycling, decomposition, and symbiotic relationships that sustain plant and animal life (Averill et al. 2022, Muhammad and Saadu 2023). These processes shape soil health and wider ecosystem resilience (Mihailovič et al. 2019, Palit et al. 2022).

Microorganisms are also central to environmental sustainability. They contribute to ecosystem restoration through pollutant degradation, composting, and wastewater treatment, and they influence greenhouse gas cycling (Kumar 2018, Kang 2021, Abubakar et al. 2022). Preserving microbial diversity is therefore critical for ecosystem stability and planetary health (Cavicchioli et al. 2019, Averill et al. 2022, Muhammad and Saadu 2023).

Indigenous land-management practices have long shaped microbial ecosystems, even if they were not described in microbial terms. Practices such as rotational harvesting, agroforestry, and organic soil amendments support soil fertility and biodiversity through their effects on microbial communities. Scientific research increasingly confirms the ecological value of these approaches. For example, controlled burns can alter soil microbial composition while supporting ecosystem regeneration (Franklin et al. 2024). Long-term soil management practices such as the creation of Amazonian Dark Earths (Terra Preta; highly sustainable fertile soils created through long-term Indigenous land management practices) also show how human–microbe–soil relationships can enhance nutrient availability and support sustainable agriculture (Glaser 2007). Traditional agricultural formulations provide another example. Panchagavya, a fermentation-based preparation derived from livestock products and used in Indian farming systems, contains complex microbial communities that support plant growth and soil health (Krishnareddy et al. 2022). These examples show that land-based practices have long shaped microbial ecosystems in ways that modern microbiology is only beginning to document.

Within this context, Indigenous practices in agriculture and environmental management can also be understood as aligning with host–microbe co-evolution (Lyu et al. 2021). Low-input systems that preserve soil microbial diversity and ecological balance help shape the microbial communities associated with foods and, consequently, human exposure (Hartman et al. 2014). These practices, developed and optimized over generations, may therefore reflect microbial ecosystems that are more compatible with human physiology (Bertola et al. 2021).

Interest in plant microbiomes is also growing as agriculture faces climate and sustainability pressures. The ‘microbiome rewilding hypothesis’ proposes that restoring microbial communities lost through industrial practices, including pesticide and antibiotic use, may improve plant resilience and ecosystem health (Mills et al. 2017). This approach draws on native soils and wild plant relatives to identify beneficial microorganisms that can be reintroduced into modern agricultural systems (Raaijmakers and Kiers 2022). Microorganisms can also act as indicators of ecosystem health. Recent studies of wild plants and traditional food systems have identified distinct microbial communities shaped by local environments and cultural practices (Yin et al. 2023, Iarusso et al. 2025). These findings reinforce the value of ecosystem-specific ecological knowledge and show how Indigenous relationships with land can inform current approaches to biodiversity conservation and ecosystem management.

Together, these examples show that Indigenous microbiology is not only about cataloguing microbial diversity. It is also about understanding long-term relationships between people, land, and microorganisms, and using that understanding to support climate adaptation, soil restoration, and biodiversity conservation.

## Traditional foods and microbial heritage

Food is a key link between culture, health, and environment, especially in Indigenous communities, where harvesting and preparation practices strengthen relationships with land. Traditional diets often differ markedly from those in industrialized societies and are associated with higher nutritional quality and greater diversity of beneficial gut microorganisms (Warbrick et al. 2023, Silk et al. 2024). Studying these food systems can therefore expand our understanding of human-associated microbial diversity, particularly in populations that remain underrepresented in microbiome research (Troci et al. 2023, Silk et al. 2024).

Fermented foods are a major part of this microbial heritage. Found across Africa, Asia, the Americas, and Oceania, they reflect long histories of local adaptation, cultural practice, and reliance on available plant and animal resources (Tamang et al. 2020, Xu et al. 2022, Ramos et al. 2023). For Māori, food preparation and preservation have long been central to culture, knowledge, and survival, with fermentation remaining an important practice (Day et al. 2022). Traditional fermented foods such as toroī (mussels and Puha), kina (sea urchins), kanga kopiro (fermented maize), and tītī (muttonbird) highlight the richness of Māori food systems and their deep connections to place, resources, and tradition (Hudson et al. 2001, Whyte et al. 2001). Similar work on Inuit fermented foods shows how processing practices and storage conditions shape microbial succession and may generate probiotic candidates adapted to Arctic environments (Campbell et al. 2022). These foods matter not only because they preserve nutrients and extend shelf life, but also because they carry cultural knowledge. Traditional fermented foods are closely tied to local ingredients, intergenerational knowledge transfer, and community identity, and they often hold important roles in seasonal events and collective gatherings (Ojeda-Linares et al. 2021, Xu et al. 2022, Zannou et al. 2022). In Māori contexts, traditional foods remain central to gatherings such as poukai (annual gatherings of the Māori King Movement) and other cultural events on marae (traditional communal meeting places), where they are shared as expressions of continuity, resilience, and mātauranga Māori (traditional Māori knowledge) (Wehi et al. 2023, Glassey et al. 2023).

Fermentation also has practical importance. In many regions, fermented foods provide affordable nutrition, support food security, and contribute to livelihoods, particularly in rural communities and among women (Tamang et al. 2020, Zannou et al. 2022, Ramos et al. 2023, Malongane and Berejena 2024). Because these foods rely on local ingredients, low energy inputs, and minimal waste, they also align with broader sustainability goals (Tamang et al. 2020, Rodzi and Lee 2021).

At the same time, the knowledge that sustains these foods is vulnerable. Fermentation practices are often transmitted orally and may be poorly documented, making them susceptible to loss (Ojeda-Linares et al. 2021, Rodzi and Lee 2021). Commercialization can further detach these foods from the communities that developed them, especially when traditional knowledge is used without recognition or benefit sharing (Ojeda-Linares et al. 2021). In Aotearoa-New Zealand, colonial disruption profoundly undermined Māori food and medicinal systems by restricting access to land, mahinga kai (traditional food gathering places and practices), and māra kai (food garden), and by marginalizing rongoā (medicinal garden) and its associated practices (Hanna and Wallace 2022, Tollan et al. 2023). As a result, many contemporary Māori diets reflect globalized food systems more than traditional foodways (McKerchar et al. 2015, Mackay et al. 2022).

Even so, food remains central to Indigenous resurgence. Efforts to revitalize traditional food practices are part of broader movements for wellbeing, cultural continuity, and food sovereignty (Wehi et al. 2023, Renall and Te Morenga 2025, Smith and Hutchings 2025). Modern molecular tools now make it possible to study the microbiota involved in fermentation in far greater detail, helping document and protect microbial heritage while retaining local knowledge and control (Krishnareddy et al. 2022, Jimenez et al. 2022, Ramos et al. 2023). These approaches can also identify candidate probiotic strains and functional cultures grounded in Indigenous diets and governance systems rather than imported commercial models (Campbell et al. 2022, Jimenez et al. 2022).

Together, these examples show that traditional foods are not only sources of nutrition. They are also microbial archives, cultural practices, and living knowledge systems. Indigenous microbiology offers a way to understand these foods on their own terms, while supporting their protection, renewal, and continued relevance.

## Therapeutic practices and probiotic potentials

Indigenous knowledge has long used native plants in forms such as teas and fermented preparations for therapeutic purposes. Microbiological research is now helping explain these practices by identifying antimicrobial compounds and beneficial microorganisms in traditional formulations. Fermented foods, in particular, have attracted renewed attention because their health benefits are linked to nutrient content, live microorganisms, and fermentation by-products (Şanlier et al. 2019, Walsh et al. 2023). Studies of Indigenous medicinal plants and complex remedies also show strong antibiofilm and antimicrobial activity, including against multidrug-resistant strains, highlighting their potential relevance to efforts to address antimicrobial resistance (Ampofo et al. 2020, Rieger et al. 2024).

Fermentation can improve the bioavailability of vitamins and minerals, increase the digestibility of proteins and carbohydrates, and reduce antinutrients that limit nutrient absorption ( Walsh et al. 2023). These practices may reflect long-standing interactions between humans and beneficial microbial communities (Leeuwendaal et al. 2022). Traditional fermented foods are also valued for their probiotic potential, particularly as sources of microorganisms that support gut health, digestion, immunity, and overall wellbeing (Rodzi and Lee 2021, Xu et al. 2022, Zannou et al. 2022).

These foods may also provide a practical alternative to commercial probiotic products by introducing beneficial microorganisms through everyday diets (Malongane and Berejena 2024). Research increasingly explores their roles as sources of prebiotics and probiotics that may influence gut microbiota, gastric peptide regulation, and insulin release (Fallah et al. 2018, Balasubramanian et al. 2024). Even when microbial composition does not change substantially, fermented foods can still affect gut metabolite production through their fibres and bioactive compounds (Zhou et al. 2019). Fermentation by-products, including bioactive peptides and exopolysaccharides, can also improve food quality and have been linked with lower risk of chronic conditions such as obesity, diabetes, hypertension, and cardiovascular disease (Şanlier et al. 2019, Walsh et al. 2023).

Fermentation is also important in medicinal teas. During tea fermentation, microbial communities transform polyphenols, amino acids, and other compounds, which can increase antioxidant activity, improve sensory properties, and enhance antimicrobial effects (Huang et al. 2022, Hu et al. 2022, Masih et al. 2023). Fermented teas have also been shown to influence gut microbiota and may provide additional health benefits (Sun et al. 2024, Zhang et al. 2024).

Despite this potential, traditional fermented products still face challenges in both commercialization and therapeutic application, particularly because many specific health claims remain under-validated (Rodzi and Lee 2021, Walsh et al. 2023). At the same time, advances in sequencing and bioinformatics are improving the ability to identify health-related genes and functions in beneficial microorganisms (Walsh et al. 2023). These tools offer new ways to evaluate traditional preparations while keeping their cultural and ecological contexts in view.

Together, these examples show that Indigenous microbiology has relevance not only for documenting traditional practices but also for shaping future approaches to health, nutrition, and antimicrobial discovery.

## Conclusion

Engaging Indigenous communities in microbiological research requires scientific rigor, cultural humility, ethical care, and long-term relationships. Indigenous microbiology challenges narrow definitions of microbial science by placing microorganisms within broader ecological, cultural, and ecosystem-specific relationships. This does not reduce scientific rigor; it strengthens the questions being asked and broadens the kinds of knowledge that inform them.

The studies discussed here show that Indigenous microbiology has practical relevance across environmental management, food systems, and therapeutic research. It offers useful perspectives for sustainable agriculture, food security, and responses to antimicrobial resistance, while also reinforcing Indigenous rights, leadership, and sovereignty in research. As microbiome science continues to grow, Indigenous knowledge should not sit at the margins of the field. It should help shape its future.

## Supplementary Material

xtag024_Supplemental_File
