# Supplementary material for: Indigenous microbiology: Why Indigenous knowledge matters in microbial science
Source: FEMS Microbes. 2026 May 6;7:xtag024. doi: 10.1093/femsmc/xtag024 (PMC13178256; doi:10.1093/femsmc/xtag024)
Supplement: xtag024_Supplemental_File [file xtag024_supplemental_file.docx]

**Reviews of manuscript FEMSMC-2026-032**

**Reviewer 1: Jana Jass, Örebro University**

**Recommendation: Minor Revision**

The manuscript provides a well-articulated and compelling argument for the value of Indigenous knowledge relating to microbiology. I found both the topic and presentation very interesting. It emphasizes that we should not dismiss indigenous activities associated with microbial process just because we do not yet know how they function.

I suggest connecting more to current society trajectories.

- I would like to see a comment on the co-evolution of humans and their associated microbiome. I expect that a number of beneficial properties identified by traditional agricultural, remediation and food practices harnessing microbial processes are also associated with co-evolution of humans and the microbes they are exposed to over the many generations. Thus, these traditional indigenous practices, would by extension be healthier for us as well as more sustainable.

- Please include how this connects to current directions in many societies – which are to promote more “organic” living and eating for both health and sustainability. This can be learned from indigenous practices. Thus agriculture, remediation and food production by indigenous peoples have been perfected and optimized over many generations and this could provide novel strategies that are more sustainable and environmentally friendly.

Some minor comments.

- Although very well written, there are a few awkward sentences, so I suggest some proofreading.

- Abstract and Introduction – The authors refer to “Indigenous knowledge systems”. I am not sure what you mean with “systems”. I suggest using just “Indigenous knowledge” – which is clear. Please remove “systems” from all occasions mentioned.

- Line 16 – instead of “land”, use “environment”.

- Line 22 – “…Indigenous knowledge can deepen microbiology research while supporting Indigenous leadership and sovereignty.” I am not sure how Indigenous microbiology supports their leadership and sovereignty. I do not think it fits into the scope of the article. Remove that part of the sentence from the abstract. Can conclude with “… and contribute to developing healthy and sustainable strategies.”

- Line 42 – “place-based” is not very clear what is meant by this. Is it regional-based? habitation-based? Please find another term that is clear. It is used in several other places in the document, should be changed there as well.

- Line 54 – please define “VANISH” taxa.

- Line 83 – What is “Amazonian Dark Earth”?

- Line 91 – The “microbiome rewilding hypothesis” – if this is not your own, it requires a reference. If it is your own hypothesis – please state that.

- Line 119 – listed Māori foods – put in brackets what they are?

- Line 129 – mātauranga Māori – what does this mean? Indigenous terms and processes are not known within the microbiology community so should be defined.

**Reviewer 2: Kathleen Scott, University of South Florida**

**Recommendation: Minor Revision**

This commentary is clearly and convincingly addresses the importance of Indigenous microbiology, an underappreciated arena of existing microbiology knowledge that merits preservation and exploration. The commentary focuses on aspects of Indigenous agriculture, fermented foods, and medicine that rely on microbial communities or provide insight into treating microbial infections, providing convincing evidence that there is much here that has benefitted the Indigenous communities that developed these insights, but also could be used globally to address many of the global concerns outlined in WHO One Health.

My suggestions are quite minor and consist entirely of suggestions to define terms that are likely to be novel to the reader.

Line 119 lists fermented foods I think. It would be good to mention what these are.

Line 128, poukai, marae. Define these

Line 129 matauranga define

Line 142 define mahinga kai, mara kai, Rongoa

**Associate Editor Recommendation: Minor Revision**

**EIC Decision: Minor Revision**

**Author's Response to Decision Letter for (FEMSMC-2026-032)**

Dears Prof. Jana Jass, Prof. Breck Duerkop and Prof. Kathleen Scott

Editors-in-Chief, FEMS Microbes

I am pleased to submit on behalf of my colleagues and me the REVISED VERSION of the manuscript FEMSMC-2026-032 entitled “Indigenous microbiology: Why indigenous knowledge matters in microbial science” to be considered for publication in FEMS Microbes. The reviewers' requests were performed, and the changes are marked with "Tracked Changes" in the manuscript. Please find below the rebuttal to each comment with an indication of changes made in the revised text.

Reviewer Comments

Reviewer #1:

Comments to the Author

The manuscript provides a well-articulated and compelling argument for the value of Indigenous knowledge relating to microbiology. I found both the topic and presentation very interesting. It emphasizes that we should not dismiss indigenous activities associated with microbial process just because we do not yet know how they function.

Answer: We sincerely thank the reviewer for this positive and thoughtful feedback. We are pleased that the relevance and presentation of the topic were well received.

I suggest connecting more to current society trajectories.

- I would like to see a comment on the co-evolution of humans and their associated microbiome. I expect that a number of beneficial properties identified by traditional agricultural, remediation and food practices harnessing microbial processes are also associated with co-evolution of humans and the microbes they are exposed to over the many generations. Thus, these traditional indigenous practices, would by extension be healthier for us as well as more sustainable.

- Please include how this connects to current directions in many societies – which are to promote more “organic” living and eating for both health and sustainability. This can be learned from indigenous practices. Thus agriculture, remediation and food production by indigenous peoples have been perfected and optimized over many generations and this could provide novel strategies that are more sustainable and environmentally friendly.

Answer: We thank the reviewer for this insightful suggestion. We have incorporated a co-evolutionary perspective in the Introduction and integrated it across relevant sections. Please see lines 60 – 72, 105 – 110, and 196 – 197.

Some minor comments.

- Although very well written, there are a few awkward sentences, so I suggest some proofreading.

Answer: Thank you for this comment. We have carefully proofread the manuscript and revised several sentences to improve clarity and readability as suggested.

- Abstract and Introduction – The authors refer to “Indigenous knowledge systems”. I am not sure what you mean with “systems”. I suggest using just “Indigenous knowledge” – which is clear. Please remove “systems” from all occasions mentioned.

Answer: Thank you for this suggestion. We have replaced “Indigenous knowledge systems” with “Indigenous knowledge” throughout the manuscript.

- Line 16 – instead of “land”, use “environment”.

Answer: We have revised “land” to “environment” in Line 16 as recommended.

- Line 22 – “…Indigenous knowledge can deepen microbiology research while supporting Indigenous leadership and sovereignty.” I am not sure how Indigenous microbiology supports their leadership and sovereignty. I do not think it fits into the scope of the article. Remove that part of the sentence from the abstract. Can conclude with “… and contribute to developing healthy and sustainable strategies.”

Answer: Thank you for the suggestion. We have revised the sentence in the abstract by removing the reference to leadership and sovereignty, as recommended.

- Line 42 – “place-based” is not very clear what is meant by this. Is it regional-based? habitation-based? Please find another term that is clear. It is used in several other places in the document, should be changed there as well.

Answer: Thank you for the comment. We have replaced “place-based” for “ecosystem-specific” throughout the manuscript to improve clarity.

- Line 54 – please define “VANISH” taxa.

Answer: We have added the definition of “VANISH” taxa in the manuscript to clarify the term for readers.

- Line 83 – What is “Amazonian Dark Earth”?

Answer: We have included the definition in the manuscript.

- Line 91 – The “microbiome rewilding hypothesis” – if this is not your own, it requires a reference. If it is your own hypothesis – please state that.

Answer: Thank you for this comment. The appropriate reference has been included in the revised manuscript.

- Line 119 – listed Māori foods – put in brackets what they are?

Answer: We thank the reviewer for this valuable suggestion. We have revised Line 119 to briefly explain each fermented food, improving clarity for readers unfamiliar with these items.

- Line 129 – mātauranga Māori – what does this mean? Indigenous terms and processes are not known within the microbiology community so should be defined.

Answer: We have revised the sentence to briefly explain the term mātauranga Māori.

Reviewer #2:

Comments to the Author

This commentary is clearly and convincingly addresses the importance of Indigenous microbiology, an underappreciated arena of existing microbiology knowledge that merits preservation and exploration. The commentary focuses on aspects of Indigenous agriculture, fermented foods, and medicine that rely on microbial communities or provide insight into treating microbial infections, providing convincing evidence that there is much here that has benefitted the Indigenous communities that developed these insights, but also could be used globally to address many of the global concerns outlined in WHO One Health.

My suggestions are quite minor and consist entirely of suggestions to define terms that are likely to be novel to the reader.

Answer: We sincerely thank the reviewer for the positive and thoughtful evaluation of our manuscript. We also appreciate the helpful suggestions regarding the clarification of terms. In response, we have revised the manuscript to include brief definitions where needed, improving accessibility for a broader readership.

Line 119 lists fermented foods I think. It would be good to mention what these are.

Answer: We thank the reviewer for this valuable suggestion. We have revised Line 119 to briefly explain each fermented food, improving clarity for readers unfamiliar with these items.

Line 128, poukai, marae. Define these

Answer: Thank you for the suggestion. We have revised the sentence to briefly explain the terms poukai and marae, for clarity.

Line 129 matauranga define

Answer: We have revised the sentence to briefly explain the term mātauranga Māori.

Line 142 define mahinga kai, mara kai, Rongoa

Answer: We have revised the sentence to briefly define the terms as suggested.

**Reviews of manuscript FEMSMC-2026-032.R1**

**Reviewer 1: Jana Jass, Örebro University**

**Recommendation: Accept**

I am satisfied that all of my comments have been addressed.

**Reviewer 2: Kathleen Scott, University of South Florida**

**Recommendation: Accept**

The authors have addressed my comments

**Associate Editor Recommendation: Accept**

**EIC Decision: Accept**
